# SENESCENCE NEW FRONTIERS - SENNET CONSORTIUM

**DOI:** 10.1093/geroni/igad104.1553

**Published:** 2023-12-21

**Authors:** Laura Niedernhofer, Viviana Perez Montes

## Abstract

Senescence is a cell fate triggered by stress, activating signaling cascades that stably arrest the cell cycle, thereby preventing mutagenesis and cancer. Senescent cells are critical for wound healing and other physiological processes, during which they secrete a host of factors that remodel tissue and attract immune cells, which are responsible for removing the senescent cells. However, with age and immune dysfunction, senescent cells accumulate and are established to play a causal role in aging and most age-related diseases, largely through their secretome that drives chronic sterile inflammation. Senescent cells have proven tractable as drug targets, potentially affording novel geroscience approaches to treat a variety of age-related diseases, including Alzheimer’s disease. To optimize this opportunity much more must be learned about senescent cells. This is a challenge as there are no specific biomarkers to detect or isolate senescent cells. Furthermore, senescent cells are anticipated to be highly heterogeneous based on cell lineage, the cause of senescence, their secretome, and tissue context. This challenge sparked the creation of the SenNet Consortium, an NIH Common Fund effort involving hundreds of scientists across the globe charged with creating a 4D atlas of senescent cells in 18 healthy human tissues across human lifespan, using mice and perturbations to inform the mapping. The speakers in this session will define the goals of SenNet, the scientific opportunities and challenges, the novel technologies applied, and the anticipated deliverables including large public datasets and innovative search and visualization tools.

